# Motivational and psychological variables related to unethical uses of artificial intelligence across multiple life domains (academics and online interactions)

**DOI:** 10.1371/journal.pone.0352419

**Published:** 2026-07-01

**Authors:** Kyle J. Messick, Ash Bass, Matthew Fulmer, Justine Williams, Jade Anderson, Mattie Wolff, Hope Taylor, Eli Wright, Graceyn Yonce, Blanca Aranda

**Affiliations:** USCB Social and Cultural Psychology Laboratory, University of South Carolina Beaufort, Bluffton, South Carolina, United States of America; University of Science and Technology of Fujairah, YEMEN

## Abstract

Past studies about unethical uses of generative artificial intelligence (AI) have focused within a specific life domain (e.g., how AI is used for cheating in academics). An online survey was conducted to investigate how AI users utilized AI across multiple domains, how unethical behaviors in one domain related to behaviors in others, and which motivational and psychological variables corresponded with those uses. The goal was to help create a foundational understanding of how people use AI more broadly, including what the largest motivations for unethical use are and if unethical use had psychological consequences for the user. Creating this foundation can help develop successful interventions for unethical AI use. Findings supported that performing potentially unethical behaviors in one area of life (e.g., academics) were positively related to unethical behaviors in another area of life (e.g., social media interactions). Using AI unethically was positively related to knowing that the use was unethical. Unethical AI use was not related to intrinsic or extrinsic motivation, but was positively related to results pressure (prioritizing outcomes), external pressure (unethical use is normative and unlikely to be penalized), and time pressure (saving time by completing a task more quickly). Unethical AI use was also positively related to a desire for social media popularity and narcissism. Unethical uses of AI were not related to self-esteem, self-efficacy, or loneliness, but were positively related to satisfaction with life, making social comparisons, internalizing the perspective of others about their own body, prioritizing physical appearance over other attributes, and comparing one’s physical appearance with others. This study provided evidence that despite ethical misuse, generative AI is being used across multiple domains of life and is largely associated with positive consequences for the user. Unethical AI use was applied across multiple areas of life and was associated with variables related to elevating status, achieving good outcomes, behaving within broader cultural norms, and saving time. Future applications could explore if a more wholistic approach that targets motivations is more effective than a domain-specific approach when attempting to create interventions to curb unethical AI use.

## Introduction

The increased prevalence of artificial intelligence (AI) in daily life has made it a high-priority interest area across many disciplines. In particular, the use of generative AI, a model of machine learning that is trained on existing data so that it can create new data similar to what it was trained on, has drawn attention for the ease of which it enables unscrupulous uses including cheating in academics and creating deepfake videos that appear real. The emergence of this type of AI has led to many questions about the ethics of its use [1] and other criticisms, including the lessening of critical thinking skills [2]. The focus of our study is to identify and explore some of the major motivational variables and psychological correlates of ethically questionable uses of AI and similar technologies across the domains of academics and online interactions. It is the hope that this broad, integrated approach spanning multiple domains will lay the groundwork for an expansive understanding of the role of AI in human behavior. Most studies focus on the use of generative AI in a specific domain of life, such as academics, but this study is novel in that it explores how AI is used by individuals across multiple areas of life. This has important implications for not only understanding how people use AI, but also how interventions for unethical use that are domain-specific are unlikely to sufficiently address behaviors applied across multiple areas of life. For example, disciplining students for using AI to cheat on assignments may not be effective if there is a broader behavior rooted in social norms where they are using AI to ‘cheat’ across multiple areas of life (e.g., using AI to fabricate photos of themselves to increase their popularity on social media platforms or to ‘catfish’ people on dating platforms). Interventions would need to target the broader behavior, rather than specific applications of it. Previous studies have not investigated broader AI uses across life domains, so this could be important for understanding if unethical AI use is domain-specific or if it is generalizable across domains, and if so, for understanding the motivations behind unethical use within and across domains. The resulting findings can serve as early groundwork for creating a more comprehensive theoretical framework that may help ascertain when and why people use AI and what consequences AI use may have for the self. As social, culture, judicial, and industry movements attempt to address unethical uses of AI, it may be fundamentally important that interventions apply across life domains and target underlying motivations, rather than continuing with current domain-specific interventions that may be ineffective due to their narrow focus. Specific focal points include investigating behaviors related to academic and writing dishonesty (e.g., AI plagiarism) and digitally manipulating or generating photos of the self for use on social media platforms and dating applications. To clarify, these potential uses are often functional and positive, such as generative AI increasingly being used to create humorous memes [3] that might improve social relations. Other uses may have negative consequences for the self, like when AI is used to fabricate or alter images of the self for social media use, resulting in non-representative images that may have negative psychological consequences for the user’s self-esteem, but these consequences and their motivations have not yet been thoroughly investigated.

### Attitudes towards AI

There are broad ethical concerns regarding the sourcing of the information fed into generative AI databases. At its simplest level, information from a myriad sources is fed to an AI database to ‘teach’ the AI model. This information often generates an average from existing information to create new output based on a request, like a new image being generated based on existing images, or a new song being generated that sounds similar to previous songs. Apart from a few generative AI databases that ethically source the materials used to ‘teach’ their AI, authors and artists largely do often do not provide consent or receive compensation for their works to be harvested by the AI platform. This questionable sourcing of information has led to multiple lawsuits and concerns about the true originality of AI output in regards to written works, visual imagery/artwork, and music [4–6]. The rampant appearance of AI-generated content across many facets of life, especially without seeking it out, has been called ‘AI slop’ [7]. As dialogue about the use of AI in everyday life has increased, individuals have begun to develop attitudes towards AI. Fietta et al. [8] described how explicit and implicit attitudes towards AI often differ and demonstrated that women generally have more negative attitudes towards AI compared to men. Research [9,10] has supported that individuals who scored high in the areas of introversion, conscientiousness, and agreeableness tend to have more positive views on AI use, suggesting that personality traits may contribute to AI attitudes. Positive attitudes towards AI among those high in introversion might suggest that individuals who prefer to work in isolation might be more likely to utilize AI as a tool. Among psychology students, Gado et al. [11] found that the intention to use AI could be predicted by attitudes, perceived usefulness, social norms, and perceived knowledge about AI. The lack of understanding about AI contributes to increasing social tension about its advancements with arguments being made on both extremes: for and against AI use. Arguments endorsing AI suggest its use as benefiting humanity through automation of menial or dangerous work. Opposing that, arguments that condemn its use usually cite economic disparity resulting from automation of formerly human-performed jobs [12,13]. These arguments may extend to the use of generative AI as a tool for future scholars who might use AI to structure, write, and edit manuscripts that are submitted to journals like this one, possibly raising concerns of sterility and monotony in the writing style of future academics. Some have even claimed that AI may replace the roles of teachers and doctors [14]. Attitudes towards AI and their associated underlying personality traits may play a role in explaining different types of AI use [15,16].

### Generative AI in academics

The role of AI in academic honesty is a major concern among academics [17]. Some concern may be warranted, as students have been found to understand blatant unethical use of AI in academics, but more subtle uses of AI that contribute to academic dishonesty may be misunderstood [18]. Most current generative AI models will often prioritize task completion over task accuracy, resulting in output that ‘hallucinates’ information that is not real or factually accurate. These ‘hallucinations’ [19] can include citing sources that do not exist, or listing real authors and journals while providing fabricated information. This results in not only plagiarism, but also AI contributing to the spread of misinformation, which students might not realize that they’re doing. A study by Murray and Williams [20] found that an individual student possesses multiple different ethical perspectives concerning the use of generative AI, rather than a single prominent perspective. Perceived ease of use and usefulness influenced student acceptance of AI systems in academic settings [21]. At the time of this research, there was a lack of studies exploring how students are using AI in academia, with only some preprints beginning to emerge [22]. Additionally, uncovering the individual qualities or situations that may mitigate or promote the use of AI need investigation.

With the rise of AI and its impact on the academic field and education, educators may find themselves questioning, evaluating, and adjusting their approaches to teaching. Fears about AI have led some teachers to adopt more reactive approaches to teaching such as implementing oral assessments to prevent AI use [23]. Others proposed proactive use by having students use generative AI to construct papers and become aware of the underlying reasons for plagiarism through workshops [24]. The focus on the negative potential of generative AI in academic writing means that the positive potential for ChatGPT and other AI platforms to enhance success and participation rates of some students is largely overlooked [25]. The role of AI in academic dishonesty dominates current arguments, but there is also discussion concerning the use of AI as a tool to reach learning outcomes [20]. For example, AI can be used to improve personalized instruction, increase students’ ability to learn independently, improve learning efficiency, and help students determine their skills and prospective employment opportunities [26]. There is an increasing push for universities to develop new policies, procedures, and regulations for their students’ use of AI in order to fully consider risks and rewards, as well as attempt to detect and prevent academic dishonesty [22]. AI may also begin to change the roles of both students and teachers [27], as perceived learning goals and different routes to obtain them emerge.

Current AI arguments overlook crucial components needed to understand changes in student behavior: when and why generative AI is being used, and what consequences that use has for the user. In academics, unethical use of AI may be consistent with previous research about cheating motivations. Intrinsic motivation has been found to have an inverse relationship with academic dishonesty [28–30]. Orosz et al. [30] found no relationship between extrinsic motivation and academic dishonesty, which contrasted work by Jordan [31] who found that cheating was related to extrinsic motivation and perceiving cheating as socially normative. Cheating has been found to have a negative relationship with self-efficacy as demonstrated by Nora & Zhang [32]. Eshun et al. [33] found that personality traits and self-efficacy had conflicting relationships with academic dishonesty. Clinciu et al. [34] took a different approach by looking at specific extrinsic pressures, and how they influence academic dishonesty. ‘Results pressure’ was defined as a student’s high interest in performance and grades, maintaining a scholarship or grant, finding a good job, and hypercompetitive behavior. ‘External pressure’ encompasses a low probability of being caught or penalized for fraud, pressure to cheat from their peer group, and/or as a response to uninteresting course content or a dislike of the teacher. Both results and external pressure were found to be positively related to academic dishonesty, so those were utilized in the current study.

### Generative AI, body image concerns, and social media

Another growing area of generative AI utilization is on social networking platforms as a tool used in online interactions. Social media users can use AI to meet strategic self-presentation goals and help maintain social connections. Strategic self-presentation includes the intentional misrepresentation of the self for the purpose of displaying a preferred self-image on social media platforms [35]. AI can be applied to an image of the self to create completely new images including fictitious backgrounds, clothing, and body augmentations that do not represent the individual in real life. Image filters similarly do this [36], and allow the user to cover up blemishes, add makeup, or emphasize qualities that exaggerate them in ways that can tailor their features towards cultural beauty standards. It has been suggested that there is a greater need to understand the use of strategic self-presentation in the context of social media [37]. In response to this need, and in addition to the AI use in academia, our study looked at the motivational and psychological variables of AI use as they relate to ethically dubious uses of AI in online interactions, including digitally manipulating photos of the self, and more specifically, using AI to alter or fabricate images of the self.

Impression management is a major motive for being involved in social networking websites [38], and individuals that are highly approval-motivated are more likely to digitally edit their photos [35]. A high emphasis on self-presentation in a social media context has been associated with more mental health struggles and reduced quality of life [39]. For example, frequent use of social media websites where there is a greater perceived need for strategic self-presentation has been associated with lower levels of self-esteem and life satisfaction [39] as a result of an increased dependence on social approval [40]. In contrast, utilizing an honest self-presentation strategy has been associated with greater self-esteem [41]. A meta-analysis of 80 studies found that more social networking site (SNS) use was positively related to narcissism and loneliness, and negatively related to self-esteem [42]. Saiphoo et al. [43] conducted a meta-analytical review of studies researching the relationship between the use of SNSs and self-esteem, and they found that lower self-esteem was associated with increased SNS use.

A large part of addressing self-presentation goals comes from body image concerns. Saiphoo and Vahedi [44] conducted a meta-analytical review concerning the relationship between social media use and body image. Body image is defined as a complex, subjective perception of an individual’s physical appearance and attractiveness, including visual perceptions, behaviors, and attitudes towards the body [45,46]. Saiphoo and Vahedi [44] found that there was a small, positive relationship between social media use and body image disturbance due to the frequency in which social media users engage in appearance-based comparisons with societally-idealized bodies or aesthetics, such as being thin or muscular. Selfie editing was associated with negative self-evaluations [47]. The negative associations between body image and self-esteem were more pronounced for women than men [48]. Self-objectification has been positively associated with using photo editing to meet strategic self-presentation needs on social media [35]. Gender differences have been found, with women editing photos more frequently, resulting in more negative affect following upward social comparisons compared to men [49].

The role of AI in strategic self-presentation is just one use of AI in a social media context. Another use is meme creation. Digital memes are images or videos with variations of text that are typically used in a humorous manner [50], though usually, memes are based on a singular visual photo. There are several functions of memes, which include communicating social and political beliefs, unifying collective identities, and developing culture [50]. They can make ideas more compact, providing images in a shareable, easily processed format. A study by Huntington [51] found that a high level of agreement with the message communicated in a political meme results in decreased scrutiny of the message, and increased perceived effectiveness of the message. The acceptance of certain memes is dependent on whether the viewer relates to attitudes in the meme. Memes can be used to increase social-relatedness on social media through humorous and/or relatable images, gifs, and videos [52]. Memes are also used as a coping mechanism when experiencing stress [53–56] and in coping with depression [56]. Research by Maclean et al. [57] suggested that image sharing may have beneficial effects for most Instagram users, as individuals high in social connectedness reported lower levels of loneliness when frequently sharing images.

### The current study

Six research objectives were pursued. The first was to see if individuals that are using generative AI in unethical ways were aware of the misuse (i.e., if they knew what constituted unethical use and use it that way anyway), or if the unethical use was due to a lack of knowledge about AI. The second was to explore motivational correlates (intrinsic motivation, extrinsic motivation, results pressure, external pressure, time pressure, a desire for popularity, personality) related to potentially dishonest or ethically dubious behaviors across academics (academic dishonesty, writing dishonesty, AI plagiarism, using AI to cheat in academics) and online interactions (digitally manipulating photos of the self, using AI to create new images of the self). The third was an exploratory investigation into how the aforementioned behaviors relate to each other (e.g., Are those being dishonest in their academic endeavors also being dishonest in their online interactions?), which would provide evidence about unethical uses being domain-specific or found across domains. The fourth looked at how the dishonest or ethically dubious behaviors related to consequential psychological variables including self-esteem, self-efficacy, satisfaction with life, loneliness, objectification of the self, body image concerns, narcissism, and personality traits. The fifth goal was to explain the variance in dishonest behaviors using the motivational variables that were positively related. Finally, the sixth goal was to explore the role of creating memes with and without AI, and to see how frequency of using social media websites and meme creation relates to psychological and motivation variables. Each objective will contribute to the overall goal of understanding if unethical uses of AI are life domain-specific or applied across life domains, what related motivations for those uses are, and what psychological consequences are associated with those uses.

## Method

### Participants

Participants were recruited through posting advertisements in American college and university social media groups, and through postings on public bulletin boards across the University of South Carolina Beaufort (USCB) campus from February to May 2024. Some USCB students received extra credit points for their completion of the survey. Participants outside of the United States were removed from the sample, leaving a total of 185 participants. 59.5% of the sample identified as female, 37.3% as male, and 3.2% as nonbinary or genderfluid. Average age was 31.72 (SD = 11.99). Most of the sample were white/ Caucasian (78.4%), followed by black (8.1%), Latino (4.9%), Asian (4.3%), Native American (1.6%), or other (2.7%). Most were single and never married (53.5%), followed by married or living as (42.2%), separated/ divorced (3.8%), or widowed (.5%). Education levels differed with most having attended some college (25.4%) or had obtained a bachelor’s degree (31.9%). 41.6% identified as first-generation students. 51.9% attended college and came from a family where their parents also attended college. 69.7% identified themselves as middle economic class, followed by lower economic class (22.7%), and upper economic class (7.6%).

### Materials

Cronbach’s Alpha was used to qualify the reliability of each scale used. Measures of AI attitudes, literacy, and ethics were included to gauge the relationship between unethical uses of AI and the knowledge of and the knowledge that those uses are unethical. Attitudes towards AI were measured using the AI Attitude Scale (AIS-4) [58], a 4-item measure that is used to gauge participants’ agreement with to what extent AI will improve their life, their work, be utilized in technology that they will use, and will be positive for humanity. It was selected based on its generalizability across diverse populations [58]. The AIS-4 was found to have strong reliability (α = .89). Understanding when using AI constitutes academic dishonesty was measured with the 11 ‘AI-giarism’ items developed by Chan [18]. The scale showed strong reliability (α = .88) and had participants state to what extent they agree or disagree that a series of different uses is academic misconduct (e.g., “The student input a prompt into an AI system, copied the generated response, and submitted it to the teacher.”). Chan [18] described ‘AI-giarism’ as “the unethical practice of using artificial intelligence technology, particularly generative language models, to generate content that is plagiarized either from original human-authored work or directly from AI-generated content, without appropriate acknowledgement of the original sources or AI’s contribution” [18, p. 4]. The artificial intelligence literacy scale [59] was used to measure four dimensions of AI literacy: awareness, use, evaluation, and ethics. The measure consists of 12 questions that participants self-report the extent to which each statement describes their literacy with different aspects of AI. Awareness including familiarity with AI (e.g., “I can identify the AI technology employed in the applications and products I use.”), knowing how to use AI effectively to complete tasks (e.g., “I can skillfully use AI applications or products to help me with my daily work.”), evaluating which AI to use for a task and what its limitations are (e.g., “I can choose the most appropriate AI application or product from a variety for a particular task”), and familiarity with ethical questions surrounding AI (e.g., “I am always alert to the abuse of AI technology”). The awareness, use, and ethics subscales demonstrated unsatisfactory reliability, but the AI evaluation subscale demonstrated satisfactory reliability (α = .77). The AL literacy scale was not used for analyses due to its overall low reliability scores.

Section III of the Academic Ethics Questionnaire [34] was used to measure external and results pressure, which consists of 23 items each scored on a 5- point Likert-type scale. Each item asked participants to rate the extent to which each scenario would influence them to engage in dishonest academic behavior (e.g., “You needed to get a good grade”, “You were pressured by other students”, “You do not like the instructor”). Results pressure (α = .89) and external pressure (α = .89) both had strong reliability.

Intrinsic and extrinsic motivation was measured using the 13-item Intrinsic Motivation and Extrinsic Motivation scale (IM-EM) [60]. The IM-EM scale had participants rate the extent to which a series of statements about motivation described them (e.g., “I prefer activities that challenge my knowledge or abilities”). Reliability was strong for intrinsic motivation (α = .85) and moderate for extrinsic motivation (α = .68).

Time pressure was measured by asking participants how frequently they use artificial intelligence to save time on a task. The desire for likes/reactions on social media posts was measured by asking participants how frequently this statement described them: “I hope that my photos, videos, and/or memes get many likes/reactions.” The importance of the popularity (virality) of social media posts was measured by asking how frequently this statement described the participant: “It means a lot to me when my photos, videos, and/or memes go viral.” Participants responded with ‘never’, ‘sometimes’, or ‘frequently’ for each question.

Section II of the Academic Ethics Questionnaire [34] was used to measure academic dishonesty and writing dishonesty, which consists of 22 items. Each item asked participants how frequently they performed each behavior during their time in school or university (e.g., “Copy someone else’s assignment”). The 13-item academic dishonesty measure (α = .95) and 5-item writing dishonesty measure (α = .89) had strong reliability. Three additional items were asked from Bashir & Bala [61] that encompassed additional forms of academic dishonesty (e.g., “Pay someone else to complete homework or an exam for me”), plus three additional items about AI use (i.e., “Use artificial intelligence on an assignment without saying that you used AI”, “Use AI to write a paper”, “Use AI to cheat on an exam or assignment”).

Specific use of photo manipulation techniques for online and mobile media was measured using the Self Photo Manipulation Scale (SPM) [62]. The SPM scale is a 10-item, self-report measure with strong reliability (α = .89). Participants were asked how frequently they used various photo manipulation techniques for self-imagery shared online and through mobile devices (e.g., “Get rid of red eye”; “Whiten your teeth”). Participants were also asked to what extent they “use AI to create new images of the self” and “use AI to create a dating profile.”

Psychological variables that were measured included self-esteem, self-efficacy, satisfaction with life, loneliness, body image concern variables, narcissism, and personality traits. Self-esteem was measured using the Rosenberg Self-Esteem Scale (RSE) [63]. The RSE is a 10-item, self-report measure. Participants rated to what extent they agreed that the series of statements described the self (e.g., “I feel that I have a number of qualities”; “I wish I could have more respect for myself.”). The RSE was found to have strong reliability (α = .88).

The General Self-Efficacy Scale (GSE) [64] was used to measure self-efficacy. The GSE is a consists of ten item scale with strong reliability (α = .90) that measure the extent to which an individual believes they can perform novel or difficult tasks and cope with adversity across multiple domains of human functioning. It reflects goal-setting, persistence in the face of obstacles, and recovery from setbacks.

Satisfaction with life was measured using the 5-item Satisfaction with Life Scale [65]. Participants responded to items stating to what extent five descriptions accurately described how they felt about life (e.g., “In most ways my life is close to my ideal”, “I am satisfied with my life”). The measure had strong reliability (α = .89).

The 8-item version of the UCLA Loneliness scale (ULS-8) [66] was used to measure participants’ sensation of being separated from others. Participants responded to the items with the frequency in which they experienced similar feelings (e.g., “I lack companionship”; “I feel left out”). It had strong reliability (α = .82).

Four measures were used to evaluate social and physical comparisons, including objectification of the self. Frequency of making social comparisons was measured using the Iowa-Netherlands Comparison Orientation Measure (INCOM) [67], which had strong reliability (α = .83). The INCOM is an 11-item, self-report measure. Participants indicated to what extent they agreed with a series of statements concerning their experience with social comparisons to other people (e.g., “I often compare myself with others with respect to what I have accomplished in life.”).

The valuation of physical appearance was measured using the Self-Objectification Beliefs and Behaviors Scale (SOBB) [68]. The SOBB scale is a 14-item, self-report measure consisting of two factors. Participants rated to what extent they agreed the series of statements best described their valuation of their physical appearance (e.g., “How I look is more important to me than how I think or feel”; “I often think of how my body must look to others.”). The SOBB consists of two factors. The first factor contains 7 items concerning the internalization of the observer’s perspective of the body (e.g., “I choose specific clothing or accessories based on how they make my body appear to others.”), which had strong reliability (α = .91). The second included 7-items about prioritizing and valuing the self through physical appearance (e.g., “Looking attractive to others is more important to me than being happy with who I am inside.”), which also had strong reliability (α = .94). Participants rated to what extent they agreed the series of statements best described their valuation of their physical appearance (e.g., “How I look is more important to me than how I think or feel”; “I often think of how my body must look to others.”).

The tendency to compare one’s physical appearance to others was measured using the Physical Appearance Comparison Scale (PAC) [69]. The PAC scale is a 5-item, self-report measure. Participants rated to what extent they agreed the series of statements described their experiences concerning physical comparison to others (e.g., “At parties or other social events, I compare my physical appearance to the physical appearance of others.”). The PAC had satisfactory reliability (α = .73).

The 10-item version of the Big Five Factor Personality Inventory (BFI-10) [70] was used to measure personality as it has been found to be as predictively capable as the 44-item BFI [71]. None of the big five personality factors had satisfactory reliability scores, so the measure was not included in the analyses. Narcissism was measured using the Narcissism subscale of the Short Dark Triad [72]. The Narcissism subscale is a 13-item, self-report measure. Participants rated to what extent they agreed that a series of statements, concerning self-importance and self-admiration, were true (e.g., “People see me as a natural leader”; “I insist on getting the respect I deserve.”). It had satisfactory reliability (α = .73).

Six additional questions were included to ask about the frequency of specific behaviors related to daily social network use (i.e., how many hours per day) and frequency of meme-related behaviors (i.e., “create memes without AI”, “create memes with AI”, “post memes online”, “use memes to improve your friendships”, “use memes to improve your relationships”).

### Procedure

The survey was completed by participants online after clicking on an invitation to complete the survey through social networking advertisements or by scanning a QR code on a poster. Following an informed consent page, participants completed items about the frequency of artificial intelligence behaviors. All other measures were presented in randomized order to reduce the possibility of order effects. Participants completed demographic items and were then debriefed.

## Results

The relationship between potentially unethical uses of AI, attitude towards AI, knowing what constitutes plagiarism with AI, awareness, usage, evaluation, and ethics related to AI were explored first. No relationship was found between attitude towards AI and knowing when AI is being used in unethical ways. There was a positive relationship between being able to identify unethical uses of AI and frequency of performing potentially unethical uses of AI. This provides evidence that individuals that are using AI in unethical ways know that they are using AI unethically. See Table 1.

**Table 1:** Pearson correlations for potentially unethical uses of AI and attitudes/understanding about AI.

	Positve attitude towards AI	AI-plagiarism knowledge	Use AI on an assignment without saying that it was used	Use artificial intelligence to cheat on an exam or other homework assignment	Use AI to create new images of the self	Use AI to create a dating profile
Positive attitude towards AI		−.058	.423**	.430**	.487**	.456**
AI-plagiarism knowledge	−.058		.375**	.310**	.356**	.500**

**Correlation significant at the.01 level *Correlation significant at the.05 level.

Pearson correlations were used to investigate the relationship between motivational variables and potentially fraudulent behaviors across academics and online interactions. Positive moderate relationships were found between results pressure, external pressure, saving time, the variables related to being liked/wanting to be popular, and each of the potentially fraudulent behavior variables (academic dishonesty, writing dishonesty, plagiarizing with AI, using AI to write a paper, using AI to cheat on an exam or assignment, digitally manipulating photos of the self, using AI to generate new images of the self, and using AI to create a dating profile). Despite results pressure and external pressure having operational overlap with extrinsic motivation, extrinsic motivation was not positively related to any potentially fraudulent behaviors, with the exception of digitally manipulating photos of the self, which had a weak, positive relationship with intrinsic motivation. See Table 2.

**Table 2:** Pearson correlations for motivational variables and fraudulent behaviors.

Motivations	Academics					Online interactions		
	Academic dishonesty	Writing dishonesty	Use AI on an assignment without mentioning it	Use AI to write a paper	Use AI to cheat on an exam or assignment	Digitally manipulate photos of the self	Use AI to create new images of the self	Use AI to create a dating profile
Results pressure (Prioritize grades, maintaining scholarship, finding a good job, being competitive)	.642**	.586**	.538**	.527**	.497**	.566**	.470**	.523**
External pressure (My peers are cheating, I probably won’t get caught, and/ or I don’t find the class or teacher interesting)	.691**	.649**	.612**	.632**	.577**	.589**	.534*	.588*
Intrinsic motivation	.062	.033	.079	.019	.029	.147*	.110	.089
Extrinsic motivation	.044	.044	.096	.018	.088	.147*	.073	.101
I use AI to save time	.583**	.582**	.592**	.590**	.561**	.628**	.598**	.650**
I hope my social media posts get many likes/ reactions	.471**	.394**	.424**	.438**	.314**	.500**	.465**	.418**
It means a lot to me when my social media posts go viral	.543**	.542**	.473**	.488**	.448**	.550**	.484**	.526**

**Correlation significant at the.01 level *Correlation significant at the.05 level.

Pearson correlations provided insights into how each potentially fraudulent use of AI was related to other potentially fraudulent uses of AI. Every behavior was moderately to strongly and positively related to every other behavior. Using AI in unethical ways in one area of life is strongly related to using AI in unethical ways in other areas of life, so the behaviors do not seem to be domain-specific. See Table 3.

**Table 3:** Pearson correlations for potentially dishonest behaviors across academics and online interactions.

	Academics					Online interactions		
	Academic dishonesty	Writing dishonesty	Use AI on an assignment without mentioning it	Use AI to write a paper	Use AI to cheat on an exam or assignment	Digitally manipulate photos of the self	Use AI to create new images of the self	Use AI to create a dating profile
Academic dishonesty		.882**	.788**	.722**	.661**	.721**	.712**	.788**
Writing dishonesty	.882**		.786**	.741**	.599**	.713**	.688**	.847**
Use AI on an assignment without mentioning it	.788**	.786**		.811**	.648**	.659**	.655**	.739**
Use AI to write a paper	.722**	.741**	.811**		.669**	.665**	.654**	.718**
Use AI to cheat on an exam or assignment	.661**	.599**	.648**	.669**		.571**	.526**	.613**
Digitally manipulate photos of the self	.721**	.713**	.659**	.665**	.571**		.668**	.737**
Use AI to create new images of the self	.712**	.688**	.655**	.654**	.526**	.668**		.781**
Use AI to create a dating profile	.788**	.847**	.739**	.718**	.613**	.737**	.781**	

**Correlation significant at the.01 level *Correlation significant at the.05 level (2-tailed).

Psychological variables were explored for their potential relationships with AI behaviors using Pearson correlations. Interestingly, unethical uses did not have a negative relationship with self-esteem despite having positive weak-to-moderate relationships with objectification of the self, comparing the self to others, and narcissism. More surprisingly, using AI in unethical ways was moderately associated with greater satisfaction with life. See Table 4.

**Table 4:** Pearson correlations for potentially dishonest behaviors and psychological/ affective variables.

	Academics					Online interactions		
	Academic dishonesty	Writing dishonesty	Use AI on an assignment without mentioning it	Use AI to write a paper	Use AI to cheat on an exam or assignment	Digitally manipulate photos of the self	Use AI to create new images of the self	Use AI to create a dating profile
Self-esteem	−.064	−.034	−.036	−.028	−.012	−.063	−.001	−.016
Self-efficacy	.110	.063	.097	.053	.111	.081	.147*	.148*
Satisfaction with life	.376**	.402**	.346**	.305**	.275**	.338**	.363**	.435**
Loneliness	.069	.070	.046	.032	.092	.136	.009	.066
Social comparisons	.210**	.198**	.196**	.159*	.166*	.343**	.153*	.207**
I internalize the perspective of others about my body	.302**	.291**	.249**	.225**	.247**	.391**	.209**	.261**
I prioritize my physical appearance over other attributes	.695**	.738**	.649**	.615**	.594**	.644**	.646**	.755**
I frequently compare my physical appearance to others	.315**	.352**	.260**	.218**	.150*	.473**	.200**	.322**
Narcissism	.373**	.344**	.330**	.283**	.280**	.321**	.313**	.379**

**Correlation significant at the.01 level *Correlation significant at the.05 level (2-tailed).

Regression analyses used motivational variables to explain the variance in academic dishonesty, cheating on an exam or assignment, and digitally manipulating photos of the self. 56% of variance in academic dishonesty was explained by the motivational variables, F(3,181) = 76.36, R^2^ = .56, p < .001. All three motivational factors were significant predictors: results pressure (β = .18, t = 2.03, p < .05), external pressure (β = .38, t = 4.13, p < .001), time pressure (β = .30, t = 5.24, p < .001).

Motivational variables explained 43% of the variance in using artificial intelligence to cheat on an exam or assignment, F(3,181) = 45.18, R^2^ = .43, p < .001. External pressure (β = .38, t = 3.62, p < .001) and time pressure were significant predicators (β = .36, t = 5.46, p < .001).

Digitally manipulating photos of the self was predicted using results pressure, external pressure, time pressure, a desire for likes on social media, and a desire to go viral on social media. The motivational variables accounted for 58% of the variance in manipulating photos of the self, F(5,179) = 45.71, R^2^ = .58, p < .001. Results pressure (β = .25, t = 2.84, p < .01), time pressure (β = .30, t = 4.94, p < .001), a desire for likes (β = .20, t = 3.26, p = .001), and a desire to go viral (β = .17, t = 2.60, p = .01) were all significant predicators.

Pearson correlations were used to look at the relationship between creating memes (with and without AI), the number of hours per day the participant used social media, the desire to get likes/reactions on post/memes, relationship functions of posting memes, meaning derived from posts/memes going viral, and psychological variables (self-esteem, self-efficacy, satisfaction with life, loneliness, comparing the self to others, internalizing the perspective of others about one’s own body, prioritizing physical appearance over other attributes, comparing physical appearance, narcissism). See Table 5. In terms of meme creation, the associations were similar regardless of the memes being created with or without AI, with the exception of satisfaction with life, comparing the self to others, and comparing physical appearance to others, which were positively related to creating memes with AI, but not creating memes without AI. The number of hours spent on social media had a negative relationship with satisfaction with life and a positive relationship with comparing the self to others. Creating memes (with or without AI) was positively associated with the desire to get likes/reactions, deriving meaning from posts going viral, prioritizing physical appearance over other attributes, narcissism, and using those memes to improve relationships.

**Table 5:** Pearson correlations related to memes, social media use, and psychological variables.

	Create memes without AI (frequency)	Create memes with AI (frequency)	Post memes online (frequency)	Social media use (frequency)	Use memes to improve friendships	Use memes to improve relationships	I hope my posts get many likes/ reactions	It means a lot to me when my posts go viral
Create memes without AI		.402**	.436**	−.014	.469**	.511**	.374**	.354**
Create memes with AI	.402**		.486**	−.008	.322**	.332**	.423**	.426**
Post memes online	.436**	.486**		−.033	.360**	.461**	.438**	.356**
Social media use	−.014	−.008	−.033		.120	.084	.119	.022
Use memes to improve friendships	.469**	.322**	.360**	.120		.826**	.264**	.234**
Use memes to improve relationships	.511**	.332**	.461**	.084	.826**		.308**	.239**
Desire for likes/reactions	.374**	.423**	.438**	.119	.264**	.308**		.568**
Meaning from own viral posts	.354**	.426**	.356**	.022	.234**	.239**	.568**	
Self-esteem	−.043	−.061	−.020	.050	−.099	−.019	.079	−.003
Self-efficacy	.102	.143	.121	−.052	.134	.089	.134	.010
Satisfaction with life	.139	.317**	.183*	−.192**	.143	.175*	.261**	.234**
Loneliness	.124	.019	.040	.008	.117	.045	.017	.091
Social comparisons	.105	.181**	.130	.173**	.297**	.262**	.274**	.145**
I internalize the perspective of others about my body	.143	.207**	.128	.081	.314**	.268**	.286**	.214**
I prioritize my physical appearance over other attributes	.260**	.584**	.258**	−.026	.222**	.234**	.404***	.483**
I frequently compared my physical appearance to others	.115	.274**	.137	.044	.289**	.266**	.211*	.220**
Narcissism	.190**	.279**	.177*	.094	.131	.159*	.343**	.262**

**Correlation significant at the.01 level *Correlation significant at the.05 level (2-tailed).

## Discussion

This exploratory study revealed that performing potentially unethical behaviors using artificial intelligence in one area of life (e.g., academics) was positively related to frequency of performing unethical behaviors in another area of life (e.g., social media interactions). This provides some of the first evidence that unethical uses of AI are not strictly domain-specific and may be part of a broader array of related behaviors that span multiple areas of life. This may have important implications as interventions and regulations are attempted to curb those behaviors. Rather than addressing specific areas for unethical AI use like academics – a more wholistic, generalizable approach may be needed. Generative AI is a new tool available to everyone, and that tool is being applied in similar ways across different areas of life. Although much of the dialogue in academia has been on unethical uses of generative AI to cheat, we have supported that the application is far broader than that. When it comes to unethical uses of AI, we looked at understanding AI ethics, associated behaviors, related motivational variables for each behavior, and if there were psychological associations with those potentially unethical uses.

It is important to note that participants were largely aware of what constituted unethical AI use while using AI in unethical ways, as there was a positive relationship between self-reported unethical uses and accurately identifying unethical use. The misuse of AI for academic dishonesty or to portray oneself dishonestly in online interactions does not appear to be, on average, unintentional or without awareness about the ethical implications.

Intrinsic and extrinsic motivation were not found to be related to academic dishonesty or cheating with AI, which partially conflicts with past research [30,31]. This could reflect an issue with the particular extrinsic/intrinsic scale used, as results and external pressure should be encompassed by extrinsic motivation, but both were found to be related to academic dishonesty and cheating with AI whereas extrinsic/intrinsic measure were not. Unlike Nora and Zhang [32], we did not find that academic dishonesty had a relationship with self-efficacy. Consistent with Clinciu et al. [34], we found that results pressure and external pressure were positively related to academic dishonesty, and we were able to extend that further to dishonest uses of AI and digital manipulation of photos of the self. Regression analyses provided further evidence that dishonest behaviors, including dishonest behaviors that utilize generative AI, seem to be heavily motivated by external and results pressure (e.g., maintaining performance and grades, finding a good job, being hypercompetitive, low perceived probability of being caught, pressure to cheat from peers, cheating being a social norm, cheating when the task or material is uninteresting to the user) and saving time on a task. The motivational contributors to dishonest AI usage seem to primarily include external factors and not internal factors such as self-esteem issues, low self-efficacy, or loneliness, which were all found to be unrelated.

People that are more approval-motivated have been found to be more likely to digitally alter photos of themselves [35], which is consistent with the outcome of our study. We found that photo manipulation and using AI to fabricate photos of the self were positively related to desiring social media likes/reactions and it being very meaningful to the user when their social media posts gain a lot of attention from others. This may also explain why ethically dubious uses of AI were associated with greater satisfaction with life. They enabled the user to meet some of their extrinsic goals for being successful, liked, and elevating status. All of the ethically questionable behaviors were positively related to making social comparisons, internalizing the objectification of the self, and prioritizing one’s physical appearance over other attributes. Social and physical appearance and the desire to be viewed in a positive light could mean that variables related to strategic self-representation play a key role in explaining unethical uses of AI. Future studies could explore more factors related to self-presentation and generative AI use and focus on using an experimental approach to determine directionality and causality. This is inconsistent with the work of Skogen et al. [73] who found that a high emphasis on self-presentation in social media was negatively related to quality of life. Generative AI might be adding a new mediator to that relationship. Yu et al. [41] found that an honest self-presentation strategy was associated with greater self-esteem, but we found no relationship between self-esteem and unethical uses of AI, including when AI was used to create fictitious images of the self.

Consistent with previous research [74], we found that digitally altering images of the self was positively associated with narcissism. We extended this further and found that narcissism was also related to using AI to create digitally altered images of the self and dishonest uses of AI in academics, cheating (more broadly) in academics, and was related to other online uses, including using AI to create a dating profile. Photo editing was also positively associated with body image concerns, which is consistent with research from McGovern et al. [75], but we expanded these findings to specifically encompass the use of AI in photo editing and dishonest behaviors in academia.

We also included an exploratory investigation into the use of memes. Consistent with previous research demonstrating that memes play a role in coping with stress [53–56,76], we found that creating memes (with AI) and posting them frequently was associated with greater satisfaction with life, and that the memes were often being used to improve social relations. Inconsistent with Maclean et al. [57], we did not find that sharing memes was inversely related to loneliness, as we found no relationship. Creating memes using artificial intelligence was positively associated with making social comparisons, internalizing sociocultural standards about physical attractiveness, and making more physical comparisons with others, but those relationships were not found when creating memes without AI. Further research is needed to understand the individual differences that underly different types of ethical and unethical uses of AI.

The study has multiple potential limitations. Recruiting through convenience sampling primarily from university campuses and online platforms may limit generalizability due to the higher education attainment and younger age of the sample, but otherwise the sample looked demographically similar to the U.S. population. A future study should put a greater focus on investigating older adults and adolescents, and pursuing a larger sample size may increase its representativeness and generalizability. The focus on correlative data limits the ability to identify causal directionality and differentiate between genuine and spurious relationships, so a future study should explore these relationships experimentally. This study may be limited by the focus on self-report data about unethical behaviors that could have been influenced by social desirability concerns. Under-reporting of undesirable behaviors may mean that the relationships reported may be even stronger. An experimental study or one that uses implicit measures could concretize the relationship further. The broad and somewhat interpretative descriptions of the behaviors (e.g., “Use AI to create a dating profile”) mean that a future study could look into more specific behaviors and provide further insight into the specific ways AI is used and for what purpose, which will help with operationalizing ethical versus unethical use of generative AI, particularly in online interactions. For example, when an individual fabricates an image of themselves using generative AI (e.g., as a football player when they have never played football), a follow-up study could provide insight into how these fabricated images are being used, whether for humor and entertainment, or to create an idealized version of themselves to gain social status in online environments. The role of personality in AI use also needs further investigation. The insufficient reliability of the ten-item Five Factor Personality measure meant that the measure was inappropriate for use in our analyses, so a future study should explore AI uses across life domains with a larger version of the Five Factor inventory that is more likely to have stronger reliability compared to the two-item factor measures used in the current study. Personality may play an important role in the relationship between AI behaviors, motivations, and psychological consequences that the current study was inadequate in exploring.

A large portion of the variance in academic dishonesty, using AI to cheat, and manipulating photos of the self was explained by motivational factors, with external pressure and results pressure being the largest predictors. It could be that that obtaining a desirable outcome and adhering to norms about artificial intelligence use are prioritized over the ethical issues with their use, despite participants communicating awareness that they were using AI in unethical ways. As cultural norms and societal pressures have increasingly emphasized standardized testing as the means to obtain positive academic and career outcomes, the process of legitimate and ethical learning processes may be less important than guaranteeing specific outcomes. Similarly, consistent with Chen et al. [35], the desire for being one’s genuine self may be less important than creating an inauthentic self that meets strategic self-presentation needs, including getting more likes and reactions on social media. Generating inauthentic images of the self using artificial intelligence could help meet those goals. Using AI and other forms of image manipulation and having a desire to receive many likes/reactions on social media had no relationship with self-esteem or self-efficacy, but did have a positive relationship with life satisfaction. Without identifying any negative consequences for the user outside of a breach in ethics, it’s easy to understand how artificial intelligence has been propelled culturally as a key tool in meeting the user’s goals, whether that’s cheating in academics to fulfill desired outcomes, save time, and meet social norms, or creating inauthentic images of the self for posting to social media accounts to increase status, likeability, and foster relationships. The motivational factors of external pressure and results pressure may need to be addressed, particularly because both are associated with greater satisfaction with life, before any diversion from unethical AI use can take place. Generative AI may be seen by users as a fundamental tool to be successful in multiple domains of life, so this may be contributing to greater satisfaction in life despite ethical concerns. These interpretations are limited by the variables selected for this study and so future studies could continue to investigate the remaining variance in unethical AI use by exploring other potentially important variables. Causality needs to be affirmed to support both the direction and interpretation of individuals’ unethical uses of AI.

The largest takeaway from the study is that unethical uses of AI do not seem to be domain-specific. Unethical uses of AI in one area of life (e.g., academics) are positively related to unethical uses in other areas of life (e.g., social media), and all of those uses have similar relations with specific motivations and psychological consequences. As discussions continue about how generative AI transforms specific domains like learning, teaching, and academics, it is important to realize that those changes are occurring across multiple facets of life, so a more wholistic approach to addressing AI behavioral issues may be more effective than domain-specific dialogue, targeted interventions, and disciplines. Future studies should further explore how AI is being used across multiple areas of life in both ethical and unethical ways, and it is proposed that interventions are developed and tested to attempt to curb behaviors across domains instead of only within them.
